# Novel Therapies for Tuberculosis: Tuberculosis Control and its Discontents

**DOI:** 10.1371/journal.pmed.0030461

**Published:** 2006-10-31

**Authors:** Jose Luis Portero, Maria Rubio

Salomon JA et al. [1] show through mathematical models the hypothetical high impact on tuberculosis (TB) control of a new shorter treatment regimen in South-East Asia. The model assumes all the positive determinants to enhance TB control but none of the negative ones, so the model is a kind of tautology that drives to an inevitable success.

Defaulting, relapsing, and drug resistance do not have to be major problems when a TB control programme based on DOTS [directly observed treatment, short-course] strategy is well established with the current six months treatment [2]. These factors are usually cited as the main obstacles to control TB, the patients being the guilty party, but the reality is more complex: failed health systems, inequity, and poverty hamper the right to use TB services.

It is arguable that shorter regimens and new technology increase case detection by themselves. Their effect is all the greater in an environment of health inequality and scarcity, as in the high TB burden countries. It is not clear if new drugs and technology will be cheaper, fully feasible, and more effective than the current ones on the field.

Strengthening of operative health research could be the fastest way in the short term to find local solutions to use meagre financial resources [3]. Migration of local health workers looking for better living conditions could be critical for the TB programmes in the future [4]. It is likely that new tools and treatments need equal or more qualified human resources than are needed today. Lack of qualified health personnel, fast rotations in their posts, and reductions of personnel due to health sector reform policies shrink the cost effectiveness of training human resources on TB control and paradoxically could increase the necessity of more personnel for the same outcome.

On the other hand, World Health Organization indicators to evaluate TB control may not be the most appropriate to measure the reality [5]. A critical revision of current measuring tools will shed light on the success and pitfalls of TB control [6]. Changes in the fashion of global disease control priorities, rise of expenses as programmes develop, and donor fatigue should be contemplated in the long term [7].

The barriers to access TB services are caused mainly by poverty and its complex socioeconomic determinants, but this key variable is hard to represent into the mathematical models [8]. Could TB be controlled through technology despite a world of increasing poverty?
